# Towards a socially fair green transition in the EU? An analysis of the Just Transition Fund using the Multiple Streams Framework

**DOI:** 10.1057/s41295-022-00304-6

**Published:** 2022-05-23

**Authors:** Anna Kyriazi, Joan Miró

**Affiliations:** Department of Social and Political Sciences, Università degli Studi di Milano, Via Conservatorio 7, 20122 Milan, Italy

**Keywords:** Just transition, European Green Deal, Multiple Streams Framework, Climate change, European Union

## Abstract

The Just Transition Fund was introduced in 2021 as part of the European Union’s Green New Deal and aims to assuage some of the painful social consequences of the green transition. Relying on the Multiple Streams Framework, this article reconstructs the JTF’s institution. It identifies 2018–2019 as a key conjuncture in the European Union when various social, ideational and political preconditions enabling policy innovation converged. Subsequently, the need to publicly finance a just transition emerged in relation to some Eastern European states’ reluctance to work towards the 2050 climate neutrality target. After a Polish-led configuration of actors propelled the JTF onto the agenda, the von der Leyen Commission assumed the task of designing a less transparently self-serving policy instrument necessary to garner wider political support. The final JTF emerged from the interplay between two policy entrepreneurs in the context of the negotiations on the 2021–2027 European Union budget and the dislocations provoked by the COVID-19 crisis.

## Introduction

At the European Council of 12 December 2019, all European Union (EU) member states except Poland endorsed the European Green Deal (EGD), a major socio-economic restructuring plan to confront climate change. Although the EGD envisages nearly 50 initiatives to ensure the so-called green transition (European Commission 2019), three elements underpin the core of its strategy. First, in April 2021, the European Climate Law was approved. This enshrines the commitment to achieve climate neutrality (zero net emissions of greenhouse gases) by 2050 and the intermediate target of reducing net greenhouse gas emissions by 55% by 2030 compared to 1990 levels. To achieve this target, the Commission unveiled in July 2021 its ‘Fit For 55’ package of 13 policies promoting decarbonisation, ranging from the introduction of carbon-related import tariffs to the expansion of the EU Emissions Trading System (EU-ETS) and the promotion of reforestation (European Commission 2021). Second, to prepare the EU’s economy for this process, the EGD’s investment plan aims to mobilise public investment for the period 2021–2030 and help unlock private funds through EU financial instruments, notably Invest EU, by at least €1 trillion (European Commission 2020a). Third, cognisant that efforts to tackle climate change need to be socially legitimate, the EGD aims to assuage some of the painful social consequences of the green transition by creating a Just Transition Fund (JTF), which will channel €17.5 billion to the regions and sectors most affected by decarbonisation, and the Social Climate Fund (SCF), which will, according to the first Commission proposal, provide €72.2 billion to help low-income households switch to more carbon-efficient equipment. Whereas the SCF is a compensating mechanism which aims to provide income support to individuals, the JTF is focused on the employment, regional and industrial-policy consequences of the green transition, seeking to support changes in models of development that remain dependent on fossil fuels.

Adding a social dimension to the energy transition was not a given: history provides examples of ‘environmentally beneficial rapid transitions undertaken in socially regressive ways’, such as the transition from coal to gas in the UK in the 1980s (Newell and Simms 2020, p. 3). The EU, conversely, decided to move the debate beyond the focus on technological change, introducing social policy considerations and questions of redistribution into the transition equation. By building on the notion of a just transition, the EGD recognises that deep decarbonisation will only be viable if it does not (or at least, does not only) constitute an additional burden for workers, households and consumers, but rather an opportunity to generate shared and inclusive prosperity. As noted above, the task of enabling the acceleration of the green transition by rendering it socially fair falls on the JTF and the SCF.

Against this backdrop, in this article we aim to account for the creation of the JTF. Certainly, the JTF and the SCF can be read as part of a wider turn in EU policy-making towards strengthening the social dimension of the integration project (Vesan et al., 2021). Nevertheless, given that new redistributive instruments are rarely adopted in the EU due to their politically charged character, the JTF and the SCF appear as remarkable policy innovations. However, while the public policy literature on the EGD is booming (e.g. Bloomfield and Steward 2020; Dupont et al. 2020; Pérez 2021), to date, few studies have been written on either the JTF and the SCF, in the latter’s case understandably, given that its legislative process is still in its initial phase. The existing studies are primarily evaluative, assessing the policy design of the JTF (Sabato and Fronteddu 2020; Sabato et al. 2021) rather than engaging with questions of power, strategy and distribution, which form the core of our perspective. To explain the political dynamics of the policy-making process leading to the creation of the JTF, we rely on the analytical lens provided by the Multiple Streams Framework (MSF) (Kingdon 2014). We ground our analysis in diverse sources of empirical evidence, comprising EU documents, media reports and ten in-depth interviews with key actors from various backgrounds, including EU institutions, social partners and civil society organisations, who were involved in the policy-making process and/or have closely followed it.^1^

We demonstrate how climate change and just transition questions emerged on the EU’s policy agenda in the late 2010s due to significant mobilisations by diverse societal actors, chief among them social movements. Taking advantage of the temporal convergence between a disparate set of social, political and ideational developments, and against the background of some Eastern European states’ reluctance to work towards the 2050 climate neutrality target, a Polish-led coalition of actors propelled the JTF initiative into the agenda-setting phase of the policy process. In reaction to these pressures, the incoming von der Leyen Commission took it upon itself to build the necessary consensus to adopt the JTF. The Commission re-designed the fund not only to placate the distributional politics of the JTF—climate laggards supporting decarbonisation in exchange for financial compensation—but also to transform the JTF from an opportunistic instrument into a proposition that could garner broad political support. The reconfiguration of the spatiotemporal features of the original window of opportunity provoked by the outbreak of the COVID-19 crisis proved key in enabling a more ambitious JTF than initially proposed.

The article is organised following the conceptual edifice provided by the MSF. The next section presents this edifice. The following three sections discuss a series of developments in the problem, political and policy environments that converged during the 2018–2019 period, thereby opening a window of opportunity to address the issue of social justice in the green transition. “The policy-making stage” section discusses the policy-making process leading to the inter-institutional agreement on the JTF’s creation in December 2020, including the conflicts within the policy community that emerged during this process and their eventual resolution. The last section wraps up the argument.

## Analytical framework

Why should the MSF be employed to explain the adoption of the JTF? Further, how should the approach be modified to do so?

The analytical lens provided by the MSF contains five structural descriptive heuristics for organising a historical policy narrative: the three streams (problems, policies and politics), windows of opportunity and policy entrepreneurs (Kingdon 2014). The problem stream refers to social problems that potentially require public policy action; the policy stream denotes the ‘primeval soup’ of policy ideas, i.e. of several potential viable solutions that originate within communities of policy makers, experts and interest groups; and the politics stream denotes the political environment that affects policy-makers’ willingness to modify existing policies. Each stream has its own dynamics and timing. However, there are moments when compelling social dislocations or events lead the streams to converge, opening windows of opportunity for policy change. Nevertheless, policy windows are successfully exploited and policy reforms adopted only if policy entrepreneurs couple the streams. Policy entrepreneurs are understood to be agents, usually operating across multiple streams, who are involved in policy design, advocacy and/or brokering practices to push forward a policy solution. These entrepreneurs ‘could be in or out of government, in elected or appointed positions, in interest groups or research organisations. But their defining characteristic […] is their willingness to invest their resources—time, energy, reputation, and sometimes money—in the hope of a future return’ (Kingdon 2014, p. 122). In doing so, a key task of policy entrepreneurs is the articulation of issue-specific advocacy coalitions to broaden the constituency behind their preferred solution. In fact, not unlike the Advocacy Coalition Framework, the MSF explains policy change as a function of shifts in coalition structures within a policy subsystem (cf. Schmid et al. 2020, p. 113).

Whereas a fully fledged discussion of the strengths and weaknesses of the MSF is beyond the scope of this article, one aspect of the MSF is particularly suggestive for analysing our case study, while one point of criticism needs to be addressed. First, the MSF offers a holistic perspective on the policy process that is useful for identifying the intricate links between diffuse social demands and specific policy-making dynamics. By advancing an understanding of policies as products shaped by the overall correlation of forces in society and the changing configurations of hegemony therein, the MSF provides a fine-grained toolkit to organise the tumult of pressures and counter-pressures, within and outside the state, which affect the development of policy-making. The literature has so far primarily focused on the attributes of both policy entrepreneurs and the political system that influence the coupling process (Ackrill and Kay 2011). We complement this perspective by illuminating the features of the policy windows that potentially affect both agenda-setting and decision-making processes.

Critical in Kingdon’s framework is the intuition that ‘ideas have their time’. The key explanatory factor in the MSF is ‘a temporal conjunction’ of diverse processes: agenda-setting, alternative-specification and decision-making (Ackrill and Kay 2011, p. 74). However, although the temporal character of policy windows lies at the heart of the MSF, little effort has been made to theoretically unpack it (for an exception, see Howlett 1998). To this end, we draw on the insights of Kriesi et al. (2021), going back to Pierson (2004), on the implications for policy-making of the spatial–temporal structures of policy problems (and thus of policy windows, given that a policy window is always a window opened to address a concrete social problem). We suggest that a given problem can be described both by the temporal mode by which a dislocation arrives (sudden or cumulative) and by the timing of its consequences (immediate or delayed). The combination of the two aspects of time imposes constraints on the policy-making modes developed to address the problem as well as the substantive responses adopted to address it. Additionally, the pressures triggered by a social problem have a spatial dimension, which is particularly relevant in the EU context. The spatial dimension can also be considered along two axes. First, the problem may affect the member states in either an asymmetrical or symmetrical way. Second, the nature of the policy solutions may require high or low interdependencies among member states, thereby producing different incentives for policy coordination. In sum, we expect the spatial–temporal configuration of social problems to have important consequences for policy-making. We do not conceive this in a mechanistic manner: ultimately, policy actors can always manoeuvre to skilfully use time, space and the institutional context to encourage certain frames and solutions instead of others.

## The problem stream

How did the social consequences of the green transition come to be seen as a problem to be tackled by public institutions? Per the MSF, three sets of factors alert decision-makers that certain conditions warrant attention: focusing events, indicators and policy feedback (Kingdon 2014). No social condition, however, automatically becomes a political problem without being constructed as such through the naming and framing activities of interested actors. The issue of climate change, due to the slow and cumulative character of its dislocations and the delayed timing of its effects, is particularly difficult to recognise as an urgent political problem and therefore likely to generate policy procrastination. Despite this, a key inflecting conjuncture took place in the EU during the 2018–2019 period, when two parallel processes of mass mobilisation on climate change and problematisation of socially regressive policy approaches to confront it developed. The engines for these developments were led by civil society actors. Three social movements emerging almost simultaneously stood out in particular: the predominantly British Extinction Rebellion (XR), the Yellow Vests in France and the youth movement Fridays for Future (FFF).^2^

The three social movements, very different in their social bases, claims and action repertoires, highlighted different aspects of the climate challenge, but their joint and cumulative effect was to call unprecedented attention to it. XR was founded in May 2018 in the UK and FFF in August of the same year in Sweden. Their subsequent transnational expansion was stunning, with the two movements organising several massive global strikes from spring to autumn 2019. Both XR and FFF reached a level of international mass mobilisation, particularly among young cohorts, that was unprecedented in the history of green politics. Greta Thunberg, spokesperson of FFF, was invited to the 2019 United National Climate Change Summit, the 2019 World Economic Forum and the Council of the EU and the European Parliament (EP) during 2020. In November 2019, the newly elected EP adopted one of FFF’s demands when it declared a ‘climate and environment emergency’ (European Parliament 2019).

The key contribution of both movements was the re-problematisation of climate change as an ‘emergency’ rather than a long-term problem, i.e. the reframing of the ‘timing of its consequences’ (Pierson 2004, p. 90). To do so, their argumentative practices relied on scientific reports and internationally agreed benchmarks, particularly the Conference of the Parties (COP) 21 2015 Paris Agreement and the 2018 Intergovernmental Panel on Climate Change (IPCC) report. In fact, scientific assessments regarding the dangerous warming of the planet had become ever more worrying during 2018–2019. In 2018, the IPCC (2018) issued a new assessment arguing for limiting the impacts of global warming from 2 to 1.5 °C by 2030. In November 2019, the journal *Bioscience* published an article endorsed by 11,000 scientists from 153 countries echoing FFF’s rhetoric of climate emergency and called for radical socio-economic transformations (Ripple et al. 2020). The protest movements pieced together disparate focusing events (the IPCC report, the COP conference, extreme weather events, etc.) into an overarching narrative. Indeed, a feedback loop developed, both at the local and international levels, between the scientific community and the green activists (Interview 8).

XR and FFF re-energised green politics by contesting the light and often market-based policy solutions that went mainstream under the discourse of ‘ecological modernisation’ (Machin 2019) in the 1990s and 2000s. However, the logic of emergency left little room for not strictly carbon-linked agenda items, particularly the socio-economic inequalities potentially amplified in the green transition. The link between the green transition and social inequality, conversely, took centre stage with the eruption of the Yellow Vests protests, which arose in France in October 2018, sparked by the French government’s planned increase of the carbon tax. Mass demonstrations began in November 2018, drawing hundreds of thousands of participants across France. Over time, the situation morphed into a political crisis for the Macron government (Kouvelakis 2019), leading to the eventual withdrawal of the proposed tax, already passed by the National Assembly.

Reminiscent of some European trade unions’ scepticism of green policies in the early 2000s (Hampton 2015), the Yellow Vests drew attention to the interests of the ‘losers’ of the green transition. They put the question of a just transition at the top of the climate agenda, warning that the distributional consequences of the green transition could not be ignored (Tooze 2020). In both press reports and our interviews, the case of the Yellow Vests came up frequently as a cautionary tale: a warning that those devising energy transition policies ignore social repercussions at their peril (Interviews 2, 4 and 5).^3^

## The politics stream

The second stream concerns the political context in which the policy process is embedded. It includes the so-called European political mood: the balance of power among organised interests and key events within the government.

**Fig. 1:**
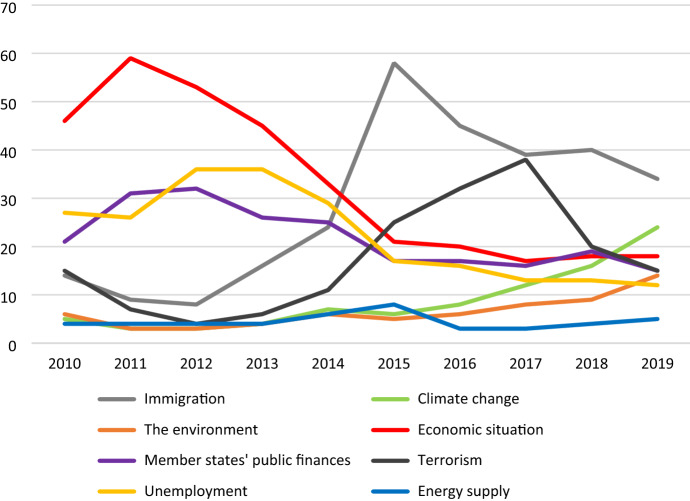
Most important issues facing the EU: selected issues, 2010–2019. Source: Standard Eurobarometer, Autumn waves. Note: The wording of the question is “What do you think are the two most important issues facing the EU at the moment?”

In the realm of public opinion, the issues of climate change and the environment became important concerns among EU citizens by 2019, when the JTF was proposed (Fig. 1). Furthermore, the proportion of Europeans who viewed tackling climate change as the responsibility of the EU had grown dramatically, from around 35% in the first half of the 2010s to almost 50% by the end of the decade (Eurobarometer 2019).

Also crucial were developments on the political calendar, with the 2019 EP elections bringing about key changes for climate action. Green parties increased their vote share by three percentage points compared to the 2014 elections, although there was a noteworthy absence of Green Members of the European Parliament (MEPs) from Eastern member states, representing only three out of a total of 69 seats won. Nevertheless, securing a share of 10% of seats increased the influence of the Greens/European Free Alliance (EFA) EP group in the imminent appointments of top EU officials. This goes a long way in explaining why the then Commission President-designate Ursula von der Leyen made the EGD a central piece of her election campaign.

The EU’s policy heritage in the environmental field also played a role. The EU has acted as an agenda setter of global climate action since the early 1990s and has consistently been at the forefront of the UN COP process, influencing its institutional design and building coalitions to push for its set goals (Parker et al. 2017). The EU adopted a comprehensive climate change strategy as early as 1992, when it endorsed the goal of limiting global warming to 2 degrees Celsius. Furthermore, in 2008 it adopted ‘the world’s most ambitious climate legislative package’ (Parker et al. 2017, p. 240). Regarding the issue of a just transition specifically, in 2017 the EU established the Platform for Coal Regions in Transition to bring together public and private stakeholders from transitioning regions and provide an institutionalised forum to share best practices. The Platform, which would operate in 41 regions located in 12 member states, constituted a favourable precedent to the JTF (Interview 7).

Nevertheless, there are several caveats and hindrances to the EU’s climate ambition. For starters, despite its efforts, the EU is not necessarily perceived as a global climate leader outside Europe and its record of realising its objectives in negotiations is mixed (Parker et al. 2017). Moreover, in the aftermath of the economic crisis, the European Commission moved towards a pattern of ‘hypocritical policy entrepreneurship’, nominally upholding its pro-environment stance but effectively giving into member states’ preferences for economic recovery and less regulation in the field of the environment (Knill et al. 2020). Additionally, there have been deep divides among various actors between and within member states as well as the EU institutions regarding the desirability of stepping up the EU’s climate targets (Skovgaard 2014). Data compiled by Climate Action Network Europe (CAN 2018) regarding countries’ progress to achieve the goals of the Paris Agreement indicate large differences among European states in their degree of commitment. Poland stands out as the most challenging case: it is the fifth-most populous and the sixth-largest EU member state and the one most reliant on coal as an energy source. Since 2015, the country has been governed by the conservative-nationalist Law and Justice party, which is among the least environmentally minded governments in the EU. The need to overcome Polish resistance in the Council was a major driver of the adoption of the JTF.

## The policy stream

The ideational precondition for devising the JTF was the elaboration of a ‘just transition’ as an appealing and feasible policy idea and its diffusion within EU governance architecture. The concept was born as a trade union demand and dates back to the 1970s, when calls to reconcile workers’ needs for decent jobs and the protection of the environment first emerged (Hampton 2015). The international trade union movement has campaigned for a socially just transition since then, referencing it in high-profile declarations, such as its statement prepared for the Kyoto Convention in 1997. The European Trade Union Confederation (ETUC) embraced the idea of a just transition in the early 2000s, gradually broadening its position from a ‘defensive’ safety net perspective, focused predominantly on mitigating potential negative impacts on workers, towards a ‘proactive’ perspective, i.e. negotiating broader systemic change (Steward 2018).

Considerations regarding a just transition were included in the preamble of the 2015 Paris Agreement, and the concept took centre stage in the COP24 held in December 2018 in Katowice (Poland), when the ‘Silesia Declaration on Solidarity and Just Transition’ (UNFCCC 2018) was endorsed by more than 50 signatories, including the European Commission on behalf of the EU. The Polish Presidency of COP24 played a key role in the adoption of the Silesia Declaration, incentivised in part by the pressures coming from Polish miners’ unions (Interview 1). Another promoter of a just transition globally has been the International Labour Organization (ILO), whose 2015 *Guidelines for a Just Transition Towards Environmentally Sustainable Economies and Societies for All* have been extremely influential in diffusing just transition policy blueprints in the EU (ILO 2015) (Interview 2). The ILO Guidelines provide a non-binding policy framework and practical tool for countries to manage the transition to low carbon economies, highlighting the need for decent jobs, social dialogue, a coherent approach across policy areas and international cooperation. The Silesia Declaration identifies the ILO Guidelines as a framework to tackle the just transition concerns mentioned in the Paris Agreement.

Throughout this period, the just transition paradigm has come to condensate a threefold aim: to protect the livelihoods of those workers made redundant due to decarbonisation policies; to actively support the transition of workers towards new types of jobs; and to promote new sustainable growth models and jobs that can replace those that are lost. A key focus of just transition programmes is therefore on investments in education, skill development and training systems, with a view of endowing workers with the competences necessary to participate in the green economy and to transition from declining to expanding sectors.

**Fig. 2:**
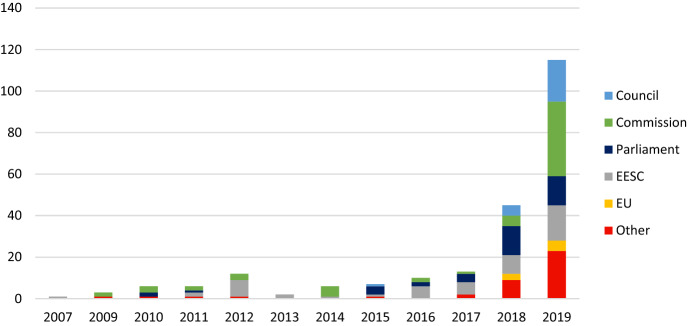
Number of EU documents mentioning “just transition”, 2007–2019. Source: Publications Office of the EU and European Council, own representation

To trace how the idea of a just transition emerged and spread over time within EU institutions, we searched for the phrase ‘just transition’ in the archives of the Publications Office of the EU.^4^ Between 2007 and 2019, as many as 226 documents mentioned this term. During most of the 2010s, references to ‘just transition’ were sporadic (Fig. 2). Even though an upward tick occurred in 2012 (linked in part to the Rio + 20 United Nations Conference on Sustainable Development), the trend reversed in the following year. References to ‘just transition’ picked up again from 2015, owing to the Paris Agreement, which was also identified as a relevant impetus for the idea in our interviews (Interview 2). A gradual and steady expansion then occurred: the number of documents mentioning ‘just transition’ increased, the institutional authors became more diverse and the policy areas viewing this concept as a concern multiplied.

Trade union activity and applied research were the earliest channels through which the idea of a just transition seeped into EU institutions and they would continue to play an important role in subsequent years. Notably, the European Economic and Social Committee (EESC), an advisory body comprising representatives of workers’ and employers’ organisations, was the main early advocate of the idea of a just transition within the EU’s institutional architecture.

Among the EU’s legislative triangle, the EP was the first to promote the idea of a just transition. In 2010, the Committee on Employment and Social Affairs (EMPL) adopted a motion for resolution titled ‘Developing the job potential of a new sustainable economy’, which the EP eventually passed. In 2017, during the revision process of the EU-ETS, the EP adopted an amendment to the Commission’s proposal for ‘Cost-effective emission reductions and low-carbon investment’, adding the following text: ‘A just Transition Fund should be established to support regions with a high share of workers in carbon-dependent sectors and a GDP per capita well below the Union average’. As EurActiv (2017) noted, ‘[i]t was the first time an EU institution proposed specific measures to address this real problem in a concrete and socially just manner, instead of vague and empty promises’. The amendment, which was not taken up by the Environmental Council, was presented by the Polish MEP Jerzy Buzek of the Civic Platform (PO) and the European People’s Party (EPP), an influential figure who has served as Prime Minister of Poland (1997–2001) as well as President of the European Parliament (2009–2012).

The Commission was relatively slow to turn its attention to the concept of a just transition and failed to incorporate it in a series of climate-related legislative proposals as late as 2018. However, whereas one third of the documents on a just transition in the year 2018 were produced by the EP, in the following year the Commission became the leading institution referencing the concept. As for the Council, it appears that it was the last major EU institution to embrace the idea of a just transition. We were only able to identify one reference to a just transition prior to 2018, namely the conclusions on the preparations for the Paris UN Climate Change Conference. Subsequently, references to a just transition were included in various Council Conclusions.

## The policy-making stage

### The opening of the policy window

A series of events occurring in November 2018 were crucial for the JTF’s inception. In preparation for the Multiannual Financial Framework (MFF) negotiations—a ‘routine political window’ (Howlett 1998, p. 500) in the EU’s political system—the EP’s Committee on Budgets (BUDG) signed off on a proposal from the Committee on Industry, Research and Energy (ITRE) allocating €4.8 billion for an Energy Transition Fund (ETF) ‘to help regions green their economies’. Buzek was again the MEP proposing the ETF and, during this initial period, he was recognised by other actors as the key policy entrepreneur pushing for the fund (Interviews 1 and 3). Buzek’s original idea was for the ETF to focus on coal regions and thus to be a targeted measure for Eastern and Central European states.

Shortly after, in December 2018, the EP held a topical debate on ‘Involving workers and citizens in a just transition to a safer planet’ (European Parliament 2018). The debate was proposed by the Socialists & Democrats (S&D) parliamentary group, with the aim of proposing ‘new specific funds for a just transition […] within the MFF’. Indicative of the extent to which the Yellow Vests protests—which had been in full swing in France at the time—were becoming an important component in the emerging narrative justifying the establishment of a transition fund, is that nine of the 22 interventions in the debate referred to them (European Parliament 2018). This suggests that the Yellow Vests contributed to recontextualising the issue of a just transition from an Eastern European concern to an EU-wide one. However, in her intervention in the EP debate, Commissioner Marianne Thyssen, despite acknowledging the need to help workers affected by the green transition, continued to advocate the creation of new funding lines within existing EU programmes, particularly the European Social Fund Plus (ESF +) and the European Adjustment Globalisation Fund (EGF), rather than instituting a new fund (European Parliament 2018). In other words, the Commission’s unresponsiveness prevented the gradual ripening of the just transition issue in the problem and policy streams from being completely coupled with the politics stream.

During 2019, the ETF/JTF proposal reappeared in discussions regarding the EU’s long-term climate objectives, as Eastern European actors demanded financial assistance to be able to implement said objectives. By June 2019, ahead of a scheduled European Council summit, the governments of several EU member states that had hitherto opposed the EU’s 2050 carbon neutrality goal (Germany, Greece, Italy and Slovakia) signalled that they were now ready to endorse it. The upcoming presidency of Finland, which had pledged to become carbon *negative* by 2050, provided additional impetus. In this context, the potential for a trade-off arose: climate laggards would sign off to the objectives in exchange for compensation funds. Nevertheless, the June 2019 European Council failed to agree on a landmark climate strategy for 2050, as the leaders of the Czech Republic, Estonia, Romania, Hungary and Poland continued to oppose it, demanding detailed pledges for funds for the countries undergoing the transition. Other member states, such as Spain, which had already undertaken substantial energy transitions in the previous years, were wary about the idea of paying others for their decarbonisation: ‘we did [it] without European help, so can you’, was their reasoning (Interview 7).

At this point, the incoming von der Leyen Commission took up the JTF proposal and set out to build a renewed policy proposal on the just transition idea, which would be less transparently self-serving from a Polish perspective and thus necessary to garner wider support.

### The legislative process

On 14 January 2020, one month after the presentation of the EGD, the Commission published its proposal for a Regulation on the JTF. The Fund was launched as one of the three pillars of the Just Transition Mechanism (JTM), alongside a dedicated just transition scheme under InvestEU and a public sector loan facility at the European Investment Bank to mobilise additional investments. Initially the JTF’s budget was set at €7.5 billion, to be complemented by the European Regional Development Fund (ERDF) and the ESF + as well as national co-financing. The total amount of investment to be mobilised under the JTM was €100 billion, with a focus ‘on the economic diversification of the territories most affected by the climate transition and the reskilling and active inclusion of their workers and jobseekers’ (European Commission 2020b). The JTF was finance projects on economic revitalisation, research activities, social support (active labour market policies) and land restoration.

While bringing EGD sceptics closer to the desired agreement on climate neutrality, the Commission’s strategy risked alienating others, especially as the debate on the JTF became enmeshed in the negotiations over the next EU budget. The ensuing discussions regarding the JTF came to be organised around four main lines of contention: the size of the fund, its conditionality, its position towards fossil fuels and nuclear energy, and the weight of its social component.

Beginning with financial size, divisions emerged between those who supported a large envelope and greater generosity and those opposing it. A related but distinct issue was whether fresh money would be found for the JTF or if, conversely, existing (cohesion) funds would be redirected towards new objectives. A heterogeneous array of actors coalesced in favour of a large JTF: conservative Eastern European governments, environmental non-governmental organisations (NGOs), trade union federations and big European companies. The ETUC had been a long-standing advocate of putting the ‘just transition’ at the core of the EU’s fight against climate change and reiterated this position during the EGD legislative process (ETUC 2019). The main European trade union in the industrial sector, IndustriAll, also complained about the size of the JTF proposed by the Commission (€7.5 billion), describing it as ‘peanuts’ (EurActiv 2020a). Equally, a range of potentially affected companies from diverse sectors also called for a bigger JTF. Indicatively, Europe’s biggest steelmaker, ArcelorMittal, warned that greater financial support was needed to meet EU carbon reduction goals without losing competitiveness to Chinese rivals (Financial Times 2020a). As this coalition of ‘strange bedfellows’ shows, the JTF had developed into something more than an exclusive request by climate inactivist Eastern European governments. Although some of these actors were pushing for a larger JTF to accelerate the green transition and others because they were concerned about this acceleration becoming excessive, a more generous JTF had become a shared demand among them.

Such mounting pressure to dedicate more resources to the JTF was met with reluctance in other member states. In reaction to the European Council’s endorsement of the JTF in December 2019, a group of net beneficiaries of cohesion funds shared their concerns that an overly large JTF might divert existing resources. Led by Portugal, Bulgaria and Spain, these countries warned against a too narrow definition of the socio-economic consequences of the green transition, arguing that their transition problems were not exclusively related to the production and use of coal, but to other polluting activities, from agriculture to plastics production. For instance, in reaction to the Commission’s proposal regarding the JTF, the Spanish Minister for Foreign Affairs, Arancha González Laya, complained that the proposal was not ‘fair’ towards Spain: ‘Spain is committed to the ecological transition, Spain supports in a very clear and determined way the JTF, but we are a little worried because we see it [as] very green but not very just for the moment’ (EurActiv 2020b). Ultimately this argument was used as a bargaining chip to defend a favourable distribution of EU funds (Interviews 7 and 9). In February 2020, the Friends of Cohesion—a permanent coordinative group among 15 member states that are net recipients of EU money—rejected the 10% cut in cohesion funds proposed by the Finnish presidency. This group, which includes both Eastern European (among them, Poland) and Southern European countries, insisted that the financing of new instruments such as the JTF could not come ‘at the expense of Cohesion Policy and Common Agricultural Policy’ (CAP) (Financial Times 2020b).

In parallel, a group of net contributors to the EU budget that came to be known as ‘the Frugals’ (the Netherlands, Finland, Sweden, Denmark and Austria) opposed any increase of the MFF, rendering the bargain process zero-sum. These governments, being committed to environmentalism, preferred to increase green expenditure while reducing the CAP and cohesion policy, as this made the national budgetary contributions more palatable to their domestic constituencies (Interview 7). In September 2019, Sweden’s deputy prime minister had already warned that her government did not want to increase its contribution to the MFF and that there were ‘substantial resources’ in the EU budget that could be channelled towards hitting climate goals (*Financial *Times 2019). The first budget proposal from the Finnish Presidency in December 2019 pushed for substantive cuts in EU cohesion funds, totalling €44 billion.

The group of Northern European countries made two further requests regarding the JTF, which put them at loggerheads with the Poland-led coalition of countries. First, the so-called Frugals argued for making JTF funding conditional upon member states’ commitment to climate neutrality by 2050. This obviously took aim at Poland, as the only member state that had not committed to this target in the 2019 December European Council. During 2020, both the German Presidency of the Council and the European Parliament proposed that 50% of a member state’s allocations could be decommitted each year until that member state pledged to achieve climate neutrality. Second, beyond the nominal commitment to the 2050 target, net contributors to the MFF were concerned about the successful implementation of decarbonisation measures, citing the weak implementation record of EU policy recommendations in some Eastern and Southern European countries.

In response, in June 2020 the Commission proposed the launch of the Just Transition Platform to assist member states in setting up their Territorial Just Transition Plans and to ensure that the funds would be directed towards the right projects and regions. However, this governance scheme was deemed insufficient by several actors, among them the European Court of Auditors (European Court of Auditors 2020) and various net contributor countries (France, the Netherlands, Austria, Denmark and Luxembourg), which bemoaned the lack of clarity in the allocation of resources and the weakness of the monitoring mechanisms. The EP also pushed for strengthening conditionality (European Parliament, 2020a), which was, expectedly, opposed by Poland, with Prime Minister Mateusz Morawiecki denouncing it as ‘political conditionality’ (EurActiv 2020c).

The third point of contention put forward by the coalition of climate-friendly net contributors concerned the energy sources to be excluded from JTF financing. This became, according to our interview data, the most salient and contentious issue. In the European Council of December 2019, Poland and the Czech Republic, in order to agree to the 2020 climate target, asked for guarantees that the green transition would not prohibit them from using natural gas and nuclear power, arguing that this was necessary to ensuring ‘energy security’ as well as respecting ‘the right of the Member States to decide on their energy mix’ (European Council 2019, p. 2). However, in its post-COVID renewed proposal on the MFF and the JTF, presented in May 2020, the Commission maintained that natural gas would be excluded from the JTF’s investments (European Commission 2020c). Shortly afterwards, eight Eastern European member states urged the EU to include natural gas projects in JTF funding, claiming that natural gas constitutes a transition fuel that is needed to shift away from coal power. Support for this position came not only from the influential lobby of European gas companies, but also the EPP, Identity and Democracy (ID), European Conservatives and Reformists (ECR) and Renew groups in the EP. To gain a sense of what was at stake for these companies, recall that between 2013 and 2020, the gas infrastructure groups received €4.049 billion in grants and loans from public authorities in the EU (Global Witness 2020). However, according to an interviewed Commission official, considerations whether to include gas were informed not only by an economic rationale, but also by a geopolitical one:

Gas has several important advantages: it is cheap and easy to deploy. But it has a terrific lobby behind it, with [a] lot of money. It has also a very interesting geopolitical implication. It is very interesting to see who defends gas, and I’m not talking of countries or political parties, but of individuals, with a lot of lobbying and geopolitics going around. This is about making Europe even more dependent on a few suppliers in Europe’s neighbourhood that are not reliable partners. (Interview 4)

Defenders of the inclusion of gas power in JTF programmes framed this as a matter of national sovereignty over energy policy and of energy-intensive consumers worried about the costs of the green transition.

On 29 June 2020, a clear majority (45 votes in favour, 17 against and 9 abstentions) in the EP’s ITRE committee voted in favour of an amendment presented by MEP Buzek recommending that gas projects should be eligible, under certain conditions, for JTF funding. Shortly afterwards, on 6 July 2020, the EP’s Committee on Regional Development (REGI)—the parliamentary committee responsible for the JTF dossier—approved the report drafted by the Greek Conservative MEP Manolis Kefalogiannis, supporting the opinion of the ITRE committee. In reaction, in July 2020, 62 environmental organisations sent a letter to the EP calling on MEPs to exclude fossil fuels from the JTF and other green-related funding (CAN 2020a). They argued that the JTF risked supporting the strategies of some member states (particularly Ireland, Italy, Greece and Hungary) to phase out coal through significant increases in fossil gas use (CAN 2020b). Nevertheless, in September 2020, the plenary of the EP ratified the inclusion of investments in natural gas (but not in nuclear energy) as eligible for JTF support.

Finally, a fourth debate that spanned the JTF negotiations concerned the relative weight of the social support component in its design. This was less important than the others, mainly being a preoccupation of the Directorate-General for Regional and Urban Policy (DG REGIO) of the Commission as well as of particularly socially minded actors such as the ETUC (Interviews 2, 4 and 7). For the DG REGIO and the European Commissioner for Cohesion and Reforms, Elisa Ferreira, there was a risk of allocating too little funding to the social component of the JTF and, specifically, to vocational training (Interview 7). For these figures, the bulk of JTF spending should be focused on social and labour market initiatives. As explained by a senior official of the Commission:

The main concern of the Commission has been to make sure that the JTF is not conceived as a pot to finance the transition proper. The JTF is there to assuage, to address the impacts of the transition, not to finance the transition. You can use the money also for energy investment, but it is clearly not its primary objective. (Interview 7)

### Towards the agreement: the reconfiguration of the policy window

The outbreak of the COVID-19 pandemic in Europe in March 2020 led to an overhaul of the MFF discussions, including those on the JTF, which until that moment had been chiefly driven by distributional struggles among member states in a context of highly asymmetrical costs and benefits. By contrast, at least two features of the new window of opportunity triggered by the COVID-19 pandemic enabled a reconfiguration of the terms of the debate. First, both the relatively symmetrical character of the economic shock among member states and the interdependent nature of the responses required to confront it enabled a robust EU-level policy response, particularly at the macroeconomic level. Second, the crisis offered an opportunity to shift social attention towards the endogenous relationships between ecological environment and society’s welfare (Dupont et al. 2020). These conditions facilitated the integration of the EGD and the JTF into the broader discussion regarding how to respond to the pandemic and to discard suggestions that the decarbonisation plans should be postponed (Dupont et al. 2020).

Thus, in late March 2020, the European Council expressed its support for advancing the green transition through the EU’s crisis response (European Council, 2020), while in April 2020 the EP called for the EGD to be at the core of the EU’s recovery strategy (European Parliament 2020b). In the Commission’s new MFF proposal presented in May 2020, the JTF increased from €7.5 to €40 billion (European Commission, 2020c). However, in the European Council of 17–21 July 2020, in which a final agreement was reached, the JTF was reduced to €17.5 billion, mainly due to the Frugals’ opposition to increasing their contributions. This figure was lower than the €40 billion proposed by the Commission in May, but higher than the €7.5 billion originally proposed in January. On balance, the COVID-dominated spring negotiations had strengthened the green component of the MFF: whereas the Commission had aimed at a share of climate expenditure of 25%, the European Council agreed that this share should be 30%.

In the EP, the JTF file was allocated to the REGI committee and was later debated in the plenary session on 16 September 2020. Amendments proposed by the EP called for increasing the JTF budget, extending the scope of support to several new areas and a derogation from excluding investments in natural gas under certain conditions (European Parliament 2020a). Significantly, in the same month, the Polish government first updated its 2040 energy strategy and soon after came to an agreement with Polish miners’ unions to phase out mines by 2049, putting the country on track to meet EU climate targets (*ΕurActiv,* 2020d).

An agreement on the JTF was reached in trilogue on 9 December 2020 (Council of the European Union 2021). In relation to the key JTF debates, the main elements of the compromise included:

1. A final size of €17.5 billion, with €7.5 billion coming from the MFF and €10 billion from the Next Generation EU instrument (in 2018 prices); the total amount of financing to be mobilised under the JTM would be €150 billion.
2. The exclusion of investments linked to fossil fuels and nuclear energy. However, this was only possible on the condition that up to 1% of ERDF financing could be used for fossil gas projects. The exchange, which at that moment went unnoticed by the public and environmental NGOs (Interview 3), attested to ‘the advantage of having different funds that can be negotiated together’, as claimed by an official from the Commission involved in the process (Interview 4).
3. Linking JTF funding to member states’ commitment to the 2050 net zero target, with only 50% of national allocation available for countries that fail to do so.
4. Ratification of the conditionality-based governance mechanism proposed by the Commission in two steps: first, pre-allocation of the funds to member states depending on three criteria (employment in carbon-intensive sectors, presence of fossil fuel extractive industries, and regional and national GDP per capita); and second, submission of Territorial Just Transition Plans to be approved by the Commission for countries to access the funds;
5. In-line with the EP proposal, broadening the eligibility scope to incorporate investments in education and social inclusion, including infrastructure for training, childcare and elderly care facilities. The JTF can also support investment to reduce energy poverty. Nevertheless, with regard to the social focus of the Territorial Just Transition Plans that the Commission sought to strengthen, this remains in the hands of member states, showing ‘the power limits of the Commission’ (Interview 7);
6. A ‘Green Rewarding Mechanism’ proposed by the EP to distribute additional resources to countries that reduce their industrial greenhouse gas emissions.

## Conclusions

When in 2018 the EU set its climate objectives for 2030, little consideration was given to social policy implications. However, the politics of climate change shifted dramatically in the year that followed: by late 2019, the EU was not only upwardly revising its climate promises, but was also taking concrete steps to tackle the just transition dimension. The initial recognition that responding to the climate change challenge would only be possible if backed by sufficient political and social majorities was formed gradually in an interplay between different sources of pressure. In the realm of society, powerful protests transformed perceptions on the available time frame to tackle decarbonisation and associated social inequalities, while dramatically increasing its political salience. In the realm of ideas, the notion of a ‘just transition’, originally born in trade union circles, was developed throughout the 2010s by European epistemic communities concerned with the social legitimacy of green politics. In the realm of politics, the formation of a ‘greener’ EP, the election of a new Commission, the drafting of a new MFF and the need to get Eastern European actors on-board the green agenda converged to open a window of opportunity for policy change.

Our article demonstrates how policy entrepreneurship and, particularly the Commission leadership, operates in the multi-level governance architecture of the EU. We argue that the final design of the JTF emerged through the synergistic and antagonistic interplay between two entrepreneurs. The first policy entrepreneur was a configuration mainly led by Eastern European actors operating from strategic positions within and outside the EU institutional architecture. They shrewdly rode the ‘green wave’, linking their demands for compensation with the ideas elaborated by trade unions and taking advantage of the pressure exerted by social protests at a moment of change in the political calendar. Once the von der Leyen Commission took up the JTF, it became the entrepreneur whose aim was to push through the proposal by building majorities for it. In doing so, the Commission’s actions became in part antagonistic to the first policy entrepreneur. The tremendous exogenous shock to the political environment exerted by the COVID-19 pandemic reconfigured the spatiotemporal features of the original policy window in a direction more prone to an ambitious EGD and JTF. The end result was a JTF in which the greener and more socially sensitive positions prevailed across the main contentious issues that emerged in the debate.

However, it is far from clear whether the JTF and the EGD will succeed in delivering a rapid and socially balanced net-zero transition in Europe. Concerns are related not only to the limited financial allocations of the instruments, but also to governance questions: several scholars have recently identified implementation problems in other cross-sectoral policy agendas promoted by EU institutions, such as regional cohesion (Fargion and Profeti 2016), as well as in environmental policy itself (Domorenok 2018). Indeed, some stakeholders are concerned about the inclusiveness and effectiveness of the Territorial Just Transition Plans (Interview 3). To discuss meaningfully the extent to which the JTF shapes domestic strategies and fosters policy learning, future research will need to turn to specific case studies and systematic analysis of best practice in key sectors.

## Appendix

See Table 1.

**Table 1:** Interviews

1	24 March 2021	David Bonson-Hesener	Policy advisor	Euracoal
2	31 March 2021	Anonymous	Policy advisor for climate	European Trade Union Confederation
3	31 March 2021	Elif Gunduzyeli	Senior policy coordinator	Climate Action Network Europe
4	13 March 2021	Anonymous	Official	European Commission
5	19 April 2021	Georgios Meleas	National expert	European Economic and Social Committee, Section for Economic and Monetary Union and Economic and Social Cohesion
6	23 March 2021	Henrique Baltazar	Assistant on the JTF portfolio to Pedro Marques (S&D, Portugal)	European Parliament
7	27 March 2021	Anonymous	Official	European Commission
8	14 May 2021	Jacopo Ciccoianni	Activist	Fridays For Future (Milan)
9	17 May 2021	Alexandra Marquardt	Attachée cohesion policy (former)	Permanent Representation of the Federal Republic of Germany to the European Union in Brussels
10	17 May 2021	Thomas Pickartz	Attachée cohesion policy	Permanent Representation of the Federal Republic of Germany to the European Union in Brussels

## Footnotes

1 A full list of interviewees can be found in the “Appendix”.

2 The name ‘Fridays for Future’ refers to the original protest actions of the movement, which consisted in pupils staying away from school on Fridays and demonstrating for climate action.

3 Although a faction of the Yellow Vests appeared dismissive of climate action, overall, this group did not oppose environmental action per se. Rather, it advocated the integration of social considerations into policy design and implementation (Kinniburgh 2019).

4 We retrieved these documents from the archive available online at https://op.europa.eu/en/home, downloading all the hits returned by the phrase “just transition”. Subsequently we discarded false positives. Given the low return of documents from the European Council/Council of the EU, we conducted an additional search on its official website, gathering 33 additional documents.
